# Hospitalisation Due to Community-Acquired Acute Kidney Injury and the Role of Medications: A Retrospective Audit

**DOI:** 10.3390/jcm12093347

**Published:** 2023-05-08

**Authors:** Henna Duong, Wubshet Tesfaye, Connie Van, Kamal Sud, Ronald L. Castelino

**Affiliations:** 1 School of Pharmacy, Faculty of Medicine and Health, The University of Sydney, Sydney, NSW 2006, Australia; 2 Sydney Medical School, Faculty of Medicine and Health, The University of Sydney, Sydney, NSW 2006, Australia; 3 Nepean Kidney Research Centre, Department of Renal Medicine, Nepean Hospital, Kingswood, NSW 2747, Australia; 4 Pharmacy Department, Blacktown Hospital, WSLHD, Blacktown, NSW 2148, Australia

**Keywords:** acute kidney injury, CA-AKI, adverse drug event, community-acquired acute kidney injury, nephrotoxic medications, SADMANS

## Abstract

The aim of this study is to assess the use of high-risk medications in patients with community-acquired acute kidney injury (CA-AKI) and the differences in the characteristics and outcomes of CA-AKI based on the use of these medications. This is a retrospective audit of adults (≥35 years) with CA-AKI admitted to a large tertiary care hospital over a two-year period. We investigated the prevalence of SADMANS (sulfonylureas; angiotensin converting enzyme inhibitors; diuretics; metformin; angiotensin receptor blockers; nonsteroidal anti-inflammatory drugs; and sodium glucose co-transporter 2 inhibitors) medications use in people with CA-AKI prior to hospitalisation. Outcomes including CA-AKI severity, kidney function recovery and in-hospital mortality were examined and stratified by use of SADMANS medications. The study included 329 patients, with a mean (SD) age of 75 (12) years and a 52% proportion of females, who were hospitalised with CA-AKI. Most patients (77.5%) were taking at least one regular SADMANS medication upon admission. Overall, 40% of patients (n = 132) and 41% of those on SADMANS (n = 104) had hypovolaemia or associated symptoms such as vomiting and diarrhoea during admission. Over two-thirds (68.1%) had mild AKI on admission and patients who were taking SADMANS medications were more likely to have mild AKI. Patients on SADMANS had more comorbidities and a higher medication burden, but there were no differences in AKI severity on admission or outcomes such as length of hospitalisation, ICU admission, need for dialysis, recovery rates and mortality between the two groups. However, the high prevalence of SADMANS medications use among patients with CA-AKI indicates a potential for preventability of CA-AKI-led hospitalisations. Future studies are needed to gain better insights into the role of withholding this group of medications, especially during an acute illness.

## 1. Introduction

An acute kidney injury (AKI) is the sudden deterioration of kidney function over a period of hours to days [1]. It is clinically defined by a sudden increase in serum creatinine (SCr) or a decrease in urine output with an accumulation of metabolic wastes and/or the development of complications such as acidosis and hyperkalaemia [1,2]. Globally, an estimated one in five hospitalised patients experience an AKI and the prevalence within the community setting is likely to be far greater [3]. Two thirds of AKI episodes are suspected to have occurred in the community setting (community-acquired AKI; CA-AKI) with over one third considered potentially preventable [4,5]. Regardless of the setting, even milder instances of AKI have been linked to the development of chronic kidney disease (CKD), subsequent progression to kidney failure and increased mortality [5,6].

The aetiology of CA-AKI remains complex and largely uncertain, but medications have often been suspected to be an exacerbating factor, particularly when patients are acutely unwell with a volume-depleting illness or infection [6,7,8]. As an extrinsic factor, deliberate withholding of nephrotoxic medication usage in the community during an acute illness has the potential to reduce the occurrence of CA-AKI. The acronym SADMANS; sulfonylureas; angiotensin converting enzyme inhibitors (ACEi); diuretics; metformin; angiotensin receptor blockers (ARB); nonsteroidal anti-inflammatory drugs (NSAID); and sodium glucose co-transporter 2 inhibitors (SGLT2i), has been developed as an aid for healthcare professionals to identify medications that could potentially cause harm during an acute illness [9,10] as use of these medications can increase the risk of development of AKI [7]. However, it is important to note that the SADMANS group of medications are also widely used in the community because of their beneficial effects, especially in patients with CKD, diabetes, hypertension and heart failure and can have significant benefits in terms of managing symptoms, reducing disease progression and mortality rates [1,11].

To minimise the potential harm of SADMANS medications, several Australian and international guidelines recommend withholding these medications during an acute illness [1,9,10,12]. Interventions trialled in the primary care setting include patient-focused educational programmes communicating the risk of AKI and self-management advice for individuals to temporarily stop specific medications when acutely unwell [12,13]. However, recommendations to withhold SADMANS medications during an acute illness are based on expert consensus with limited evidence either demonstrating a positive impact of withholding SADMANS on clinical outcomes associated with CA-AKI or on worsening of the medical condition that these drugs were originally prescribed for [12,13,14]. Given this background, the objectives of this study were to determine the extent of SADMANS medication usage in people hospitalised with CA-AKI and the clinical characteristics, severity and outcomes of CA-AKI in patients on SADMANS versus those not prescribed SADMANS. We hypothesised that in patients who developed CA-AKI, taking SADMANS medications was associated with poorer clinical outcomes.

## 2. Materials and Methods

### 2.1. Study Design, Participants and Setting

This retrospective cross-sectional audit included a collection of data from patients admitted to a tertiary care university teaching hospital in metropolitan New South Wales, Australia between 1 January 2020 and 31 December 2021 (24 months). Patients aged ≥35 years, who were admitted through the emergency department with an unplanned hospital stay for at least 24 h and who received an internal hospital coding of “acute kidney injury”, “acute or chronic renal failure” or “acute renal impairment” were eligible for inclusion. Patients were excluded if they developed AKI during their hospital admission, received regular renal replacement therapy or had a history of kidney transplantation. Patients with no recorded SCr value within the first 48 h of their hospitalisation or those who had a separate, distinct hospital admission in the 48 h prior to the admission of interest were also excluded. This study was approved by the Western Sydney Local Health District (WSLHD) Human Research Ethics Committee (2019/PID14671).

### 2.2. Data Collection

Patient data, including demographics, diagnosed medical conditions and regular medications were obtained retrospectively through the electronic medical records (eMR). Relevant pre-existing comorbidities were categorised based on the International Classification for Diseases (ICD-10) to support the calculation of the Charlson comorbidity index (CCI). Relevant pathology results and clinical information including presenting symptoms, admitting medical team, time to nephrology consult if any, suspected AKI aetiology and clinical outcomes were recorded.

### 2.3. Outcomes

The primary outcome of this study was the proportion of CA-AKI patients using SADMANS medications. Differences in clinical characteristics of patients prescribed the SADMANS group of medications versus those not prescribed SADMANS, severity of AKI and outcomes including length of hospital stay, rate of ICU admission, rate of dialysis during admission and at discharge, extent of recovery of kidney function, rate of readmission in the subsequent 6 months after index hospitalisation and mortality, were also reported.

### 2.4. Definitions

Three different methods were used to determine the presence and severity of CA-AKI. If a patient had a known baseline SCr value, CA-AKI and AKI staging were determined using the Kidney Disease Improving Global Outcome (KDIGO) AKI criteria on the basis of the first admission SCr compared to the baseline SCr [1]. Patients who developed AKI in the subsequent 48 h after admission were identified to have hospital-acquired AKI (HA-AKI) and were excluded from the study. The Murray and Duff criteria identify patients already recovering from an AKI upon admission to hospital based on a decrease from the SCr value on admission by at least 33% in the subsequent 7 days [15]. For patients with an unknown baseline SCr value, the Murray and Duff AKI criteria were used to retrospectively identify and stage CA-AKI. The Murray and Duff criteria were also applied for patients who had an SCr value within the past 12 months but did not meet KDIGO AKI criteria. For patients who did not meet either the KDIGO criteria or the Murray and Duff criteria, CA-AKI was confirmed through the review of medical officer notes.

AKI diagnosis based on a urine output criterion was not applied because of the retrospective nature of this study, as it was poorly reported within the first 48 h of admission and the reliability of recorded values could not be ascertained. For all patients who were determined to have CA-AKI-related admission as per the KDIGO AKI criteria, the extent of kidney function recovery was defined based on a change in the KDIGO AKI staging during the patients’ transition from admission to discharge. “Complete recovery” was the absence of AKI per the KDIGO criteria at discharge. “Partial recovery” was defined as a fall in AKI stage. No recovery was concluded if “complete” or “partial” recovery criteria were not met. The extent of recovery could not be reported for patients who were confirmed to have CA-AKI through the review of medical officer notes or Murray and Duff criteria.

### 2.5. Statistical Analysis

Statistical analysis was conducted using IBM SPSS Statistics (Version 28) [16]. Descriptive statistics on continuous variables were reported using mean and standard deviation (SD) if these were normally distributed and as the median and interquartile range (IQR) if the distribution was skewed. Categorical variables were reported as the frequency and proportion. Baseline characteristics and outcomes of patients taking SADMANS medications were compared to those not taking SADMANS medications using parametric and nonparametric tests. Independent sample t-tests or Mann–Whitney U tests were used for continuous variables as appropriate depending on the normality of data distribution for individual variables. For categorical variables, Chi-squared or Fisher-Exact tests were used depending on the observation counts within individual categories. Statistical significance was set at a two-sided *p*-value of 0.05 for all analyses.

## 3. Results

A total of 569 patients had an unplanned admission and received a relevant hospital-coded diagnosis of AKI within the two-year study period. Of these, 329 patients were determined to have community-acquired AKI and were included in the final analyses. The baseline demographics and clinical characteristics of the study cohort are presented in Table 1.

Most patients (n = 255; 77.5%) admitted with CA-AKI were taking at least one regular SADMANS medication on admission and 54% (n = 179) were on two or more SADMANS medications. Nine patients (2.7%) were taking five SADMANS medications, which was the maximum number of SADMANS being taken in our cohort. Over 40% of all patients (n = 132) and 41% of patients on SADMANS (n = 104) presented with hypovolaemia or associated symptoms such as vomiting and diarrhoea on admission. The most common SADMANS category were diuretics (n = 205; 36.7%) followed by ARBs (n = 129; 23%), metformin (n = 81; 14.5%), ACEi (n = 62; 11%), sulfonylureas (n = 46; 8%), NSAIDs (n = 25; 4.5%) and SGLT-2 inhibitor (n = 11; 2%). The most common SADMANS medication taken was furosemide (n = 116; 21%), followed by metformin (n = 81; 14.5%), hydrochlorothiazide (n = 46; 8%), gliclazide (n = 45; 8%) and perindopril (n = 42; 7.5%).

Patients on SADMANS medications took a higher number of regular medications (mean of 9.7 (SD = 4.00) versus 4.19 (SD = 3.49), *p* < 0.001), had a higher Charlson Comorbidity Index (CCI) score (mean of 2.71 (SD = 1.69) versus 2.08 (SD = 1.65), *p* = 0.004) and were more likely to have hypertension (*p* < 0.001), diabetes (*p* < 0.001) and a history of prior admission within 6 months preceding the index admission (*p* = 0.032). However, there was no difference in the proportion of patients with CKD between the two groups (*p* = 0.215).

There was no significant difference in terms of baseline, admission or in-hospital peak SCr values between patients taking SADMANS and those who did not.

**Figure 1:**
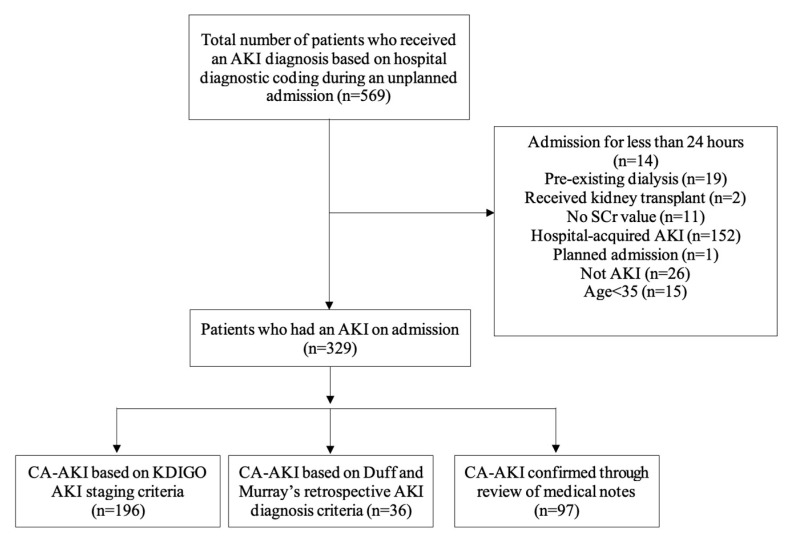
Flow diagram of study cohort inclusion.

Over half of all patients (n = 196; 59.6%) had a baseline value recorded within 12 months prior to their admission, which enabled AKI staging using the KDIGO’s AKI criteria, while 36 patients (10.9%) were staged using the Duff and Murray criteria (Figure 1). A total of 97 patients (29.5%) could not be staged on admission but were confirmed to have CA-AKI from documentation in their discharge summaries. Of patients whose stage of AKI could be determined, over two thirds (n = 158; 68%) had mild AKI (stage 1) on admission, with patients who were taking SADMANS medications more likely to have mild AKI (stage 1) than those not taking SADMANS medications (*p* = 0.04). However, there was no statistically significant difference in the overall distribution of AKI severity on admission by staging on admission (*p* = 0.41).

AKI aetiology was not reported for 35.5% (n = 117) of all patients. Prerenal causes were implicated in around half of all patients (54%; n = 179). A SADMANS medication was identified as either being a contributing or primary cause of AKI in 33 patients (10%). Of patients who had a SADMANS medication as their primary cause of AKI (n = 8), furosemide was implicated in five patients. Five patients were also taking a “triple whammy combination” (concurrent use of an ACEi or ARB, with a diuretic and an NSAID).

Table 2 presents hospitalisation outcomes for patients taking versus not taking SADMANS medications. There were no statistically significant differences in terms of length of hospitalisation, rates of ICU admission, rates of dialysis during admission, rates of dialysis continued following discharge, the extent of kidney function recovery and in-hospital mortality based on the use of SADMANS medications. While patients on SADMANS had a higher readmission rate within six months of index hospitalisation (48% versus 36.5%; *p* = 0.08), this was not statistically significant between the two groups.

## 4. Discussion

This study presents findings on the clinical characteristics of patients admitted to a tertiary care hospital with CA-AKI and differences in outcomes based on the use of medications that may increase the risk of AKI. This is the first Australian study determining the use of SADMANS medications in people with CA-AKI and exploring outcomes of patients taking SADMANS medications as a group since the endorsement of the Sick Day Plan to prevent AKI in the community by Kidney Health Australia [9,10]. Overall, this study attempts to address the need for studies exploring contributing factors for CA-AKI and measures that can be taken in the community to prevent AKI [17]. Consistent with previously published prospective and retrospective studies examining CA-AKI, our study cohort consisted largely of older adults with multiple chronic conditions [8,18].

Our findings indicate two thirds of patients admitted to hospital due to CA-AKI were taking at least one SADMANS medication and over half of them took two or more of these medications. The high prevalence of SADMANS use, particularly diuretics and ACEi, is comparable with previous findings reporting on the use of nephrotoxic medications in patients with CA-AKI [6,8,18,19]. A major retrospective review of AKI hospitalisations conducted in Taiwan reported that over 50% of patients (n = 3111) with CA-AKI had a prescription for an antihypertensive medication and 18% of patients with CA-AKI were taking an antiglycaemia agent [8]. Our study identified 33 patients whose SADMANS medication(s) were a contributing factor to the development of their AKI. Anecdotally, among patients who had a definite drug-induced CA-AKI, furosemide was the most implicated medication. Large pharmacovigilance studies reflect the significant consequences of the SADMANS drug classes as diuretics were the second most implicated medication (18.5%), followed by renin–angiotensin system inhibitors (16.3%), for causing drug-induced AKI [19]. Additionally, five patients were on a “triple whammy” regimen prior to their hospitalisation. It is apparent that SADMANS drugs can lead to patient harm and the high prevalence of SADMANS use found in our study highlights the clear rationale for ongoing optimisation of patients’ medication regimen. This could be achieved through regular medication review, management programmes and proactive monitoring of adverse effects in the primary care setting to prevent the development of CA-AKI.

Additionally, our study demonstrates a substantial proportion of patients taking SADMANS (41%) who presented with complaints associated with hypovolaemia and volume loss. Prior studies assessing the preventability of CA-AKI determined that the majority of potentially avoidable AKI required the adjustment of medications such as an ACEi/ARBs during an intercurrent infection or dehydration [20]. These findings emphasise that CA-AKI is often a multifactorial event and the combination of medications and disease states must be considered in CA-AKI prevention. These patients may particularly benefit from self-management interventions currently being trialled that emphasise early patient recognition of acute illness, early withholding of SADMANS medications and early contact with a relevant healthcare professional such as a GP or pharmacist to reduce the risk of developing CA-AKI [21,22,23].

While being on SADMANS medications prior to hospitalisation was not significantly associated with worse outcomes such as length of hospitalisation, ICU admission, in-hospital mortality or recovery rates, people on these medications had a significantly higher medication and disease burden on admission. Chaumont et al. found that the use of ACEi/ARBs was associated with an increased risk of developing AKI and AKI-prolonged hospitalisation [24]. In contrast, a systematic review exploring the potential harms of continuing ACEi/ARBs versus mortality, rehospitalisation and changes to kidney function on discharge found there was no significant difference [14]. However, most existing studies including ours found it difficult to determine the association between SADMANS medications and AKI outcomes as there is confounding by indication [11,25]. That is, patients taking SADMANS medications are more likely to have a higher risk of adverse outcomes following an AKI because the disease states that SADMANS medications are used to treat e.g., diabetes and heart failure, independently increase the risk of adverse outcomes [26]. Finally, most patients in both the SADMANS and no SADMANS groups of our study cohort were admitted with stage 1 (mild) CA-AKI, which has a weaker association with adverse outcomes [27]. This potentially explains the lack of difference in clinical outcomes observed between our two study groups. Additional advanced analyses using larger samples and stratifying patients by the level of AKI severity may provide additional insight in this regard. However, the small representation of patients with stage 2 (moderate) and stage 3 (severe) AKI in our study cohort have precluded us from performing such inferential analysis.

Additionally, while there was no statistically significant difference in the outcomes between patients taking SADMANS versus those not, it is well established that any AKI is subsequently linked to increased risk of heart failure, chronic kidney disease and poorer long-term survival—potentially doubling the risk of death in the subsequent 5 years [28,29,30]. In our study, we also observed a trend indicating that a higher proportion of people on SADMANS were rehospitalised within six months, although it was not statistically significant. This is in line with recent reports that patients had a significantly higher risk of 30-day and 90-day all-cause readmission that increases with the number of pre-existing comorbidities [26,31]. It is crucial that patient comorbidities be properly managed following their discharge from an AKI-associated hospitalisation to prevent subsequent readmission and disease progression. Comorbidities of concern include diabetes, ischemic heart disease, congestive heart failure and CKD—mainstay indications for SADMANS medications [32]. There is substantial evidence that continued use of ACEi/ARBs following an episode of AKI may be renoprotective and lower the risk of all-cause mortality, recurrence of AKI and progression to CKD [32,33]. Thus, there exists a dichotomy in the use of SADMANS medications where they may increase the risk of AKI in the context of acute illness but for the large part have significant benefits in managing morbidities that may also place patients at risk of AKI and poorer outcomes [29]. Overall, there is a clinical imperative to maintain a nuanced balance of the benefits of SADMANS medications versus the potential risks.

Overall, our study provides preliminary evidence to understand the association between SADMANS medications use and the development of CA-AKI and patient outcomes. It forms the foundations for future studies to determine the potential risks associated with taking SADMANS medications during an acute illness. Particularly, the extensive use of the medications in people with CA-AKI underlines the need for further investigation on the individual versus the added contribution of different medications that fall under this category. Additionally, in line with multiple guidelines and primary initiatives that aim to prevent avoidable AKI incidents, our work highlights the need to explore further the role of medications in contributing to AKI and devise effective interventions for prevention. Further, the risks associated with withholding these medications during an acute illness in terms of potential worsening of the condition that these drugs were originally prescribed for also need to be quantified. Interventions could include guidance to withhold SADMANS medications when unwell and patient education to recognise the role of medications in AKI and self-manage their conditions. Acknowledging this, future studies should adopt a strong methodology to explore the impact of continuing and/or withholding SADMANS medications in broader settings where AKI can develop—including in primary care.

### Strengths and Limitations

The strengths of our study include that it is the first to report on the association of SADMANS with outcomes as one comparison group. This was in response to the growing number of interventions that broadly target multiple chronic medications summarised by the mnemonic SADMANS, to prevent AKI and related adverse events [10,12,22]. This is a key strength of our study as the existing literature is often limited to a single class of medications or broadly considers all nephrotoxic medications, including ones used in an acute treatment setting such as antibiotics, which may not be relevant when planning prevention strategies [6,8,14,25,34]. Additionally, by incorporating the Duff and Murray method to retrospectively identify CA-AKI in the instance where the KDIGO criteria could not be applied, we were able to incorporate more patients into our study cohort to reflect a more accurate number of resolving CA-AKI on hospital admission.

There are limitations that should be considered when interpreting our findings. The study was conducted at a single tertiary hospital, hence the findings may not be applicable to other settings, particularly those with different demographic characteristics. Furthermore, because of the retrospective nature of our study, there were some relevant data that could not be ascertained for all patients. Notably, defining kidney function recovery involves a clear assessment of the baseline kidney function to distinguish nonrecovery of renal function from pre-existing CKD or the progression of CKD. A baseline indication of kidney function within the 7 days prior to admission would need to be requested from the community setting [1,15]. In our study, around a quarter of patients did not have a baseline SCr recorded within 12 months prior to admission. Patients not on any SADMANS medications were more likely to not have a baseline SCr compared to those who were on one or more SADMANS medications. This impacts the accuracy of determining the incidence and severity of CA-AKI and prevents definitive conclusions from being made about the association between SADMANS medication use and recovery rates.

Finally, the limited scope of our study may affect its applicability in informing clinical decisions regarding medication management within the primary care setting. Recent studies have reported that most patients do not recall receiving advice to withhold SADMANS medications if they are acutely unwell, nor do primary care practitioners routinely recommend patients withhold SADMANS medications in times of acute illness [22,23,35]. However, we were unable to determine whether patients discontinued their medications in the community prior to their hospitalisation and it is unknown whether any discontinuation of medication would have impacted our findings. Furthermore, a large proportion of patients who developed AKI in the community, particularly if mild, may not be hospitalised or remain undetected [3,17]. Patients admitted to hospital with CA-AKI may be those with more severe AKI or other critical illnesses. Likewise, our study reports on the association between taking SADMANS medications and outcomes when a patient is hospitalised with CA-AKI. It remains uncertain if the extrapolation of outcomes from a hospital setting would translate to the community setting. Moreover, we studied patients only until 6 months after the index admission and, therefore, we were unable to report on the impact of SADMANS medications on long-term outcomes. Finally, although people with a kidney transplant also have a considerable risk of developing AKI [36], we have excluded this patient population from our study, which affects the generalisability of our findings to transplant settings.

## 5. Conclusions

We observed that a large proportion of patients with CA-AKI take SADMANS medication(s), with nearly 7 in 10 taking at least one medication from these classes. More importantly, some of these medications have contributed to AKI-led hospitalisation as evidenced by the documentation in patient records, indicating that hospitalisations with CA-AKI could be prevented in a large proportion of patients by avoiding these medications during an acute illness. Whilst hospitalised patients with CA-AKI on SADMANS had milder AKI, there were no significant differences in outcomes between people with or without SADMANS medications. Our findings were indicative of the need for more research to understand the role of such medications in AKI-led hospitalisations, particularly those with community onset. Due to the potential link between the use of SADMANS medications and the risk of AKI, especially in the context of an acute illness, there is a strong rationale for additional studies exploring quality use of medicines with the view to identify and prevent CA-AKI and hospitalisations associated with medications.

## Figures and Tables

**Table 1:** Baseline and admission characteristics of study cohort.

Characteristics	All Patients (n = 329)	SADMANS (n = 255)	No SADMANS (n = 74)	*p*-Value
Age (mean (SD))	75.00 (12.0)	75.29 (11.5)	74.02 (15.1)	0.505
Males (*N* (%))	159 (48.3)	123 (48.2)	36 (48.6)	0.950
Number of medications (mean (SD))	8.47 (4.52)	9.71 (4.00)	4.19 (3.49)	<0.001
Charlson Comorbidity Index (mean (SD))	2.57 (1.70)	2.71 (1.69)	2.08 (1.65)	0.004
Hypertension (*N* (%))	237 (72.0)	202 (79.2)	35 (47.3)	<0.001
Chronic kidney disease (*N* (%))	109 (33.1)	89 (34.9)	20 (27.0)	0.205
Diabetes (*N* (%))	156 (47.4)	142 (55.7)	14 (18.9)	<0.001
Prior hospitalisation ^a^ (*N* (%))	156 (47.4)	129 (50.6)	27 (36.5)	0.032
SCr baseline ^b^ (µmol/L (median (IQR)))	108.50 (84.00–148.00)	110.00 (84.00–147.00)	97.00 (84.00–152.00)	0.687
SCr on admission (µmol/L (median (IQR)))	175.00 (119.00–259.00)	176.00 (122.00–251.50)	170.50 (113–320.00)	0.982
Maximum SCr (µmol/L (median (IQR)))	190.00 (135.00–298.00)	190.00 (139.50–292.00)	195.00 (123.00–327.00)	0.787
AKI stage on admission (*N* (%)) ^c^				0.413
Stage 1	158 (68.1)	130 (71.4)	28 (56.0)	0.038
Stage 2	43 (18.5)	30 (16.5)	13 (26.0)	0.125
Stage 3	31 (13.4)	22 (12.1)	9 (18.0)	0.276
eGFR baseline (mL/min/1.73 m^2^ (median (IQR))) ^d^	51.00 (34.00–68.50)	50.50 (34.00–69.00)	51.00 (34.00–64.00)	
eGFR on admission (mL/min/1.73 m^2^ (median (IQR)))	29.00 (17.00–43.00)	29.00 (17.00–43.00)	24.50 (15.00–49.00)	0.787
Nephrology consultation ^e^ (*N* (%))	95 (28.9)	78 (30.6)	17 (23.0)	0.203
Days to nephrology consult (mean (SD))	1.62 (4.76)	1.69 (5.07)	1.29 (3.06)	0.673

AKI—acute kidney injury, eGFR—estimated glomerular filtration rate, SCr—serum creatinine; ^a^ within 6 months preceding the index admission; ^b^ 83 patients did not have an SCr baseline within the preceding 12 months and were not included; ^c^ 97 patients could not be staged by the KIDGO or Murray and Duff criteria and were not included; ^d^ 93 patients did not have an eGFR value and were not included. *p*-value could not be determined as eGFR of patients with normal kidneys was reported as >90 mL/min/1.73 m^2^. Exact eGFR values were unknown; ^e^ renal admissions were included in nephrology consultation count.

**Table 2:** Hospitalisation outcomes of study cohort.

Outcomes	All Patients (n = 329)	SADMANS (n = 255)	No SADMANS (n = 74)	*p*-Value
Length of hospitalisation (median (IQR))	7 (4–11)	7 (4–11)	7 (4–11)	0.813
ICU admission (n (%))	63 (19.1)	48 (18.8)	15 (20.3)	0.781
Dialysis during admission (n (%))	11 (3.3)	10 (3.9)	1 (1.4)	0.279
Dialysis on discharge (n (%))	10 (3.0)	8 (3.1)	2 (2.7)	0.848
Kidney function recovery (*N* (%)) ^a^				0.523
Complete	117 (66.1)	98 (68.1)	19 (57.6)	0.251
Partial	14 (7.9)	10 (5.5) (6.94)	4 (12.1)	0.320
No recovery	46 (26.0)	36 (25.0)	10 (30.3)	0.531
Rehospitalisation ^b^	149 (45.3)	122 (47.8)	27 (36.5)	0.084
In-hospital mortality	19 (5.8)	13 (5.1)	6 (8.1)	0.328

ICU—intensive care unit; ^a^ recovery could not be determined for 152 patients—133 patients because they did not have a baseline SCr within the preceding 12 months and 19 patients did not have a discharge SCr recorded. These patients were not included in this section; ^b^ within subsequent 6 months following index admission.

## Data Availability

The data presented in this study are available on request from the corresponding author.
